# Gravity‐Driven Assembly Dynamics of Liquid Metal Microdroplets for Functional Composite Films

**DOI:** 10.1002/smll.74498

**Published:** 2026-07-13

**Authors:** Brittan T. Wilcox, Ryan C. Rothermel, Michael D. Bartlett

**Affiliations:** ^1^ Mechanical Engineering, Soft Materials and Structures Lab Virginia Tech Blacksburg Virginia USA; ^2^ Macromolecules Innovation Institute Virginia Tech Blacksburg Virginia USA

**Keywords:** composite, electrical conductor, electronics, fabrication, liquid metal, materials science, polymer, settling, viscosity

## Abstract

Liquid metal (LM) microdroplets have emerged as versatile building blocks for soft and stretchable electronics, enabling compliant conductors, multilayer architectures, and reconfigurable systems. Among the manufacturing strategies used to structure these composites, gravitational settling in liquid‐phase polymer films offers a scalable pathway for directed assembly; however, the mechanics and kinetics governing this process remain poorly understood. Here we create an experimental platform and quantitative image‐analysis framework to measure the gravitational settling dynamics of highly packed LM microdroplets in liquid‐phase polymer resin films. By systematically varying LM microdroplets size (∼50−500 μm diameter), resin viscosity (∼100−5000 mPa s), and the geometry of the film system (∼300−1600 μm thick), we elucidate the process‐structure relationships of gravitational settling of LM in confined films. Settling time decreases with droplet diameter following an inverse‐square Stokes‐type scaling and increases linearly with resin viscosity, which dominates the assembly rate. In contrast, film and saturated layer thicknesses exert only minor influence due to confinement effects. These relationships translate directly to a multilayer assembly process for soft interlayer via fabrication, establishing a predictive framework for scalable gravity‐directed LM assembly.

## Graphical abstract

This article presents a new understanding of the gravitational settling of liquid metal microdroplets in polymer resin films. It employs an experimental and analysis platform for quantification of the process‐structure relationships to inform the dynamics of gravity‐driven assembly in soft circuit architectures.

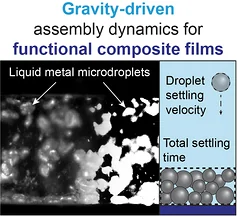

## 1 Introduction

The emergence of soft and stretchable circuits for next‐generation wearable electronics necessitates new manufacturing strategies that depart from those developed for conventional rigid circuit boards. Traditional microfabrication processes are optimized for depositing and patterning rigid metals such as copper and gold onto dimensionally stable substrates. In contrast, soft electronic systems commonly rely on composite material architectures, where micro‐ to nanoscale conductive fillers such as silver particles [1, 2, 3, 4], carbon nanotubes [5, 6, 7, 8, 9, 10], liquid metal (LM) [11, 12, 13, 14], and combinations thereof [15, 16, 17, 18, 19, 20] are dispersed within elastomeric matrices to impart both compliance and conductivity. Among these, LM is particularly attractive due to its ability to accommodate for large strain through fluidic deformation while preserving electrical and thermal functionality [21, 22, 23, 24, 25, 26]. However, the transition from rigid metal films to dispersed conductive composites fundamentally changes how conductive pathways are defined, patterned, and integrated, introducing new challenges in achieving microscale resolution, multilayer soft circuit boards, and mechanically robust circuit architectures at manufacturing‐relevant scales.

**Figure 1:**
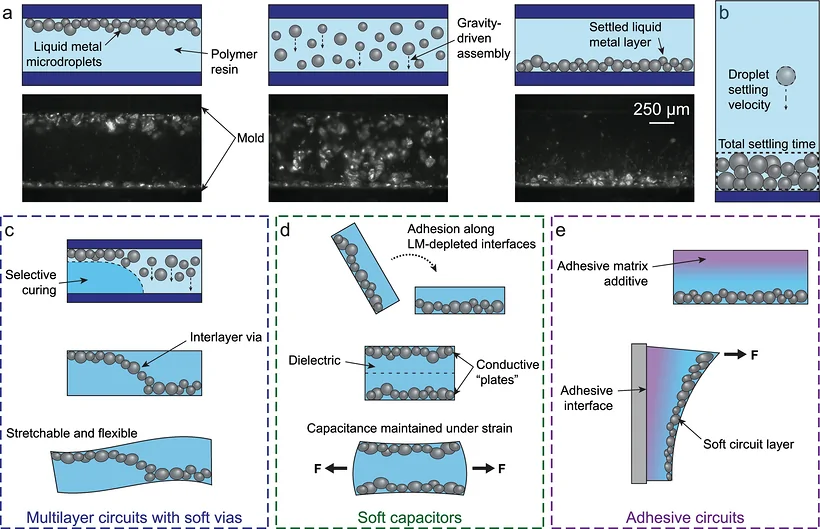
Gravity‐driven assembly dynamics of liquid metal microdroplets in polymer resin for functional composite films. (a) Gravitational settling of LM microdroplets in polymer resin films and (b) schematic of measured parameters. LM soft electronics methodologies leveraging gravitational settling of LM, including (c) fabrication of multilayer soft, stretchable, and flexible circuits with interlayer vias [47], (d) stretchable soft capacitors through bonding of two LM‐settled films [46], and (e) soft circuits with integrated adhesives [44].

A variety of methods for patterning LM into circuits have been proposed [27, 28, 29, 30, 31, 32, 33, 34], including indentation of bulk LM composites to selectively form conductors [13, 35], spray deposition of LM [36, 37, 38], and direct‐ink‐write of LM filaments [39, 40, 41] or LM composites [22, 42, 43]. Many of the methods for patterning LM in elastic substrates or matrices involve the dispersion of microdroplets of LM in liquid‐phase uncured polymer resins [15, 22, 42, 44, 45, 46]. Since the density of LM ($\rho_{LM}=6.25$ g/mL) is greater than the polymer resins employed as soft circuit substrates ($\rho_{resin}\approx 1$ g/mL), the force of gravity can cause LM to settle to the bottom of a suspension (Figure 1a,b). Prior works in LM composite films have used a wide variety of matrix materials and processing methods and have shown varied LM suspension results, from heterogeneous systems with near‐total phase separations [44, 45, 46, 47] to those creating uniform, homogeneous dispersions throughout the film [48, 49, 50]. Even in LM composite systems with the goal of homogeneous dispersions, settling presents a challenge: keeping LM suspended until the polymer matrix has gelled sufficiently to prevent movement of fillers [48, 49, 51]. Strategies to mitigate settling include careful tuning of volume fraction, droplet size, and rheological modifiers to enable curing with a uniform dispersion [49] as well as flipping LM composite films during curing so that bidirectional settling promotes a more homogeneous droplet distribution [48, 52].

Alternatively, gravitational settling of LM droplets within uncured polymer films can be strategically leveraged as a scalable manufacturing pathway, enabling directed assembly into heterogeneous and spatially organized composite architectures. When combined with a photoresin matrix and selective ultraviolet curing, settling has enabled multilayer circuits with simultaneously formed interlayer vias across the entire film that avoids sequential drilling (Figure 1c) [47], stretchable soft capacitors formed through LM phase separation of the electrode‐insulator interface (Figure 1d) [46], reversibly adhesive LM circuit surfaces through controlled LM‐depleted and adhesive rich regions (Figure 1e) [44], and LM‐hydrogel based soft conductors with enhanced electrical and mechanical properties [53]. Despite these varied demonstrations, the fundamental mechanics and kinetics governing LM settling remain underexplored, demonstrating the need for a systematic investigation to inform process design and fabrication to achieve either homogeneity or heterogeneity for future soft electronic devices.

In this work, we develop a new experimental apparatus and analysis methods to quantify the gravitational settling of highly‐packed LM microdroplets in liquid‐phase uncured polymer films. This purpose‐built image acquisition technique, alongside a video processing and analysis workflow, enables us to measure the time duration for settling to complete. Multiple parameters of interest are varied, including LM microdroplet size, film thickness, viscosity of the suspending media, and relative thickness of the LM‐saturated layer. Video motion tracking of individual LM microdroplets is also employed to determine settling velocities, supplementing the total settling time analysis. We further examine the dynamics of settling within an ultraviolet lithography‐based LM multilayer circuit fabrication method. Through this study, the process conditions and relationships for gravitational settling of LM microdroplets in films are elucidated, enabling future manufacturing process design for industrialization of LM soft circuits leveraging settled architectures.

## 2 Results and Discussion

### 2.1 Materials and Processing

**Figure 2:**
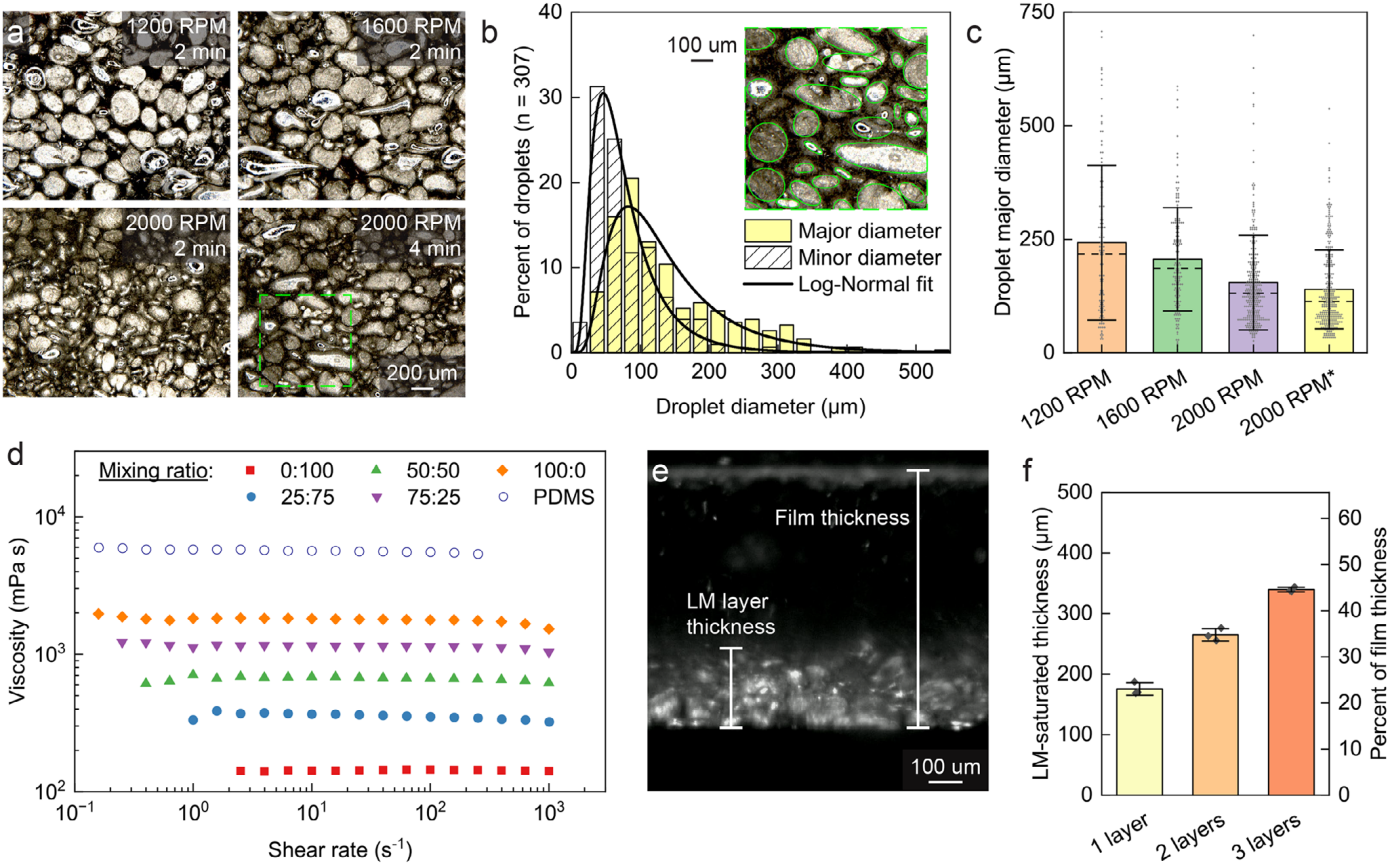
Analysis of LM microdroplet and resin material system. (a) Optical micrographs of LM microdroplets mixed at various speeds. (b) Histogram of major and minor diameters of LM microdroplets mixed at 2000 RPM for 4 min. Inset depicts a representative portion of ellipses fit onto droplets for analysis, corresponding to marked region in (a). (c) Droplet major diameters for all mixing conditions. 2000 RPM* refers to the 2000 RPM, 4 min mixing condition, all other datasets are mixed for 2 min at listed mixing speeds. (d) Viscosity of different mixing ratios of resins. Mixing ratios are listed in mass ratios of (high viscosity resin):(low viscosity diluent resin). (e) Horizontal section micrograph of settled LM‐saturated layer. (f) Thickness of settled LM‐saturated layer for varying numbers of masking film layers. Right y‐axis represents corresponding percent of overall nominal film thickness (760 μm). Data represents mean value ± s.d. (n = 3). Dashed lines in (c) represent median values.

Eutectic gallium‐indium (EGaIn) liquid metal microdroplets are dispersed in a liquid‐phase polymer resin solution through shear mixing. This results in a solution of higher‐density LM microdroplets suspended in a lower‐density polymer resin in which gravitational settling can occur. By changing the speed in shear mixing, the size of LM microdroplets can be controllably tuned. We employ mixing speeds of 1200, 1600, and 2000 RPM for 2 min, with an additional condition of 2000 RPM for 4 min, to achieve microdroplets of varying sizes (Figure 2a). The droplet size of LM is measured through optical microscopy and image analysis. Through image analysis, ellipses are fit to each droplet and the major and minor diameters are recorded (Figure 2b and Figure S1). Log‐normal fits were applied independently to the distributions for the major and minor diameters, and the statistical values for all parameters including d_10_, d_50_, and d_90_ are listed in Table S1. For a mixing speed of 1200 RPM, the largest droplets have a mean major diameter of 250 μm and minor diameter of 150 μm, with multiple outliers with diameters in excess of 600 μm. For higher mixing speeds the size of LM microdroplets decreases, with mean major diameter decreasing to 220, 150, and 140 μm for higher mixing speeds and times (Figure 2c). Additionally, tighter microdroplet distributions are achieved at mixing speeds of 1600 RPM or higher with few microdroplets in excess of 500 μm.

The viscosity of the resin in which LM is suspended was varied through mixing of a high viscosity resin and a low viscosity diluent resin. Rheological measurements were taken on mixtures ranging from entirely low‐viscosity diluent resin (0:100) to only high‐viscosity photoresin (100:0), as well as for polydimethylsiloxane (PDMS). These viscosities span a representative range of photocurable resins, from a minimum of 141 mPa s to a maximum of 1790 mPa s, with the PDMS increasing to 5580 mPa s. The resin mixtures exhibit approximately Newtonian behavior over the shear‐rate regime relevant to droplet settling, and average viscosity values were therefore calculated. The local shear rate surrounding a settling droplet can be approximated as $\dot{\gamma }∼u_{T}/d_{e}$, where $u_{T}$ is the droplet settling velocity and $d_{e}$ is the droplet diameter [54, 55]. This yields characteristic shear rates on the order of $10^{-1}-10^{2}$ s^−1^ for the conditions explored in this work.

These droplet suspensions are then made into films with varying total thicknesses and LM‐saturated layer geometries. Three total film thicknesses of 320, 760, or 1600 μm thick are employed. The thickness of the initial LM‐saturated region is also varied in fabrication; this parameter corresponds to the height of the settled LM microdroplet layer in the initial state (Figure 2e; Figure S2a–c) and is set through the use of 1, 2, or 3 layers of adhesive mask in the drawdown step of fabrication. As the drawdown process isolates a highly packed layer of settled LM microdroplets that is subsequently covered with unfilled resin, increasing the LM‐saturated layer thickness also increases the overall LM content within the film. The thinnest LM‐saturated layer thickness (1 layer) is 176 ± 10.3 μm, and additional layers add a further 87.4 ± 16.4 μm each (Figure 2f). For the 760 μm films, the LM‐saturated layer therefore occupies approximately 20%−45% of the total film thickness.

### 2.2 Gravitational Settling Characterization

**Figure 3:**
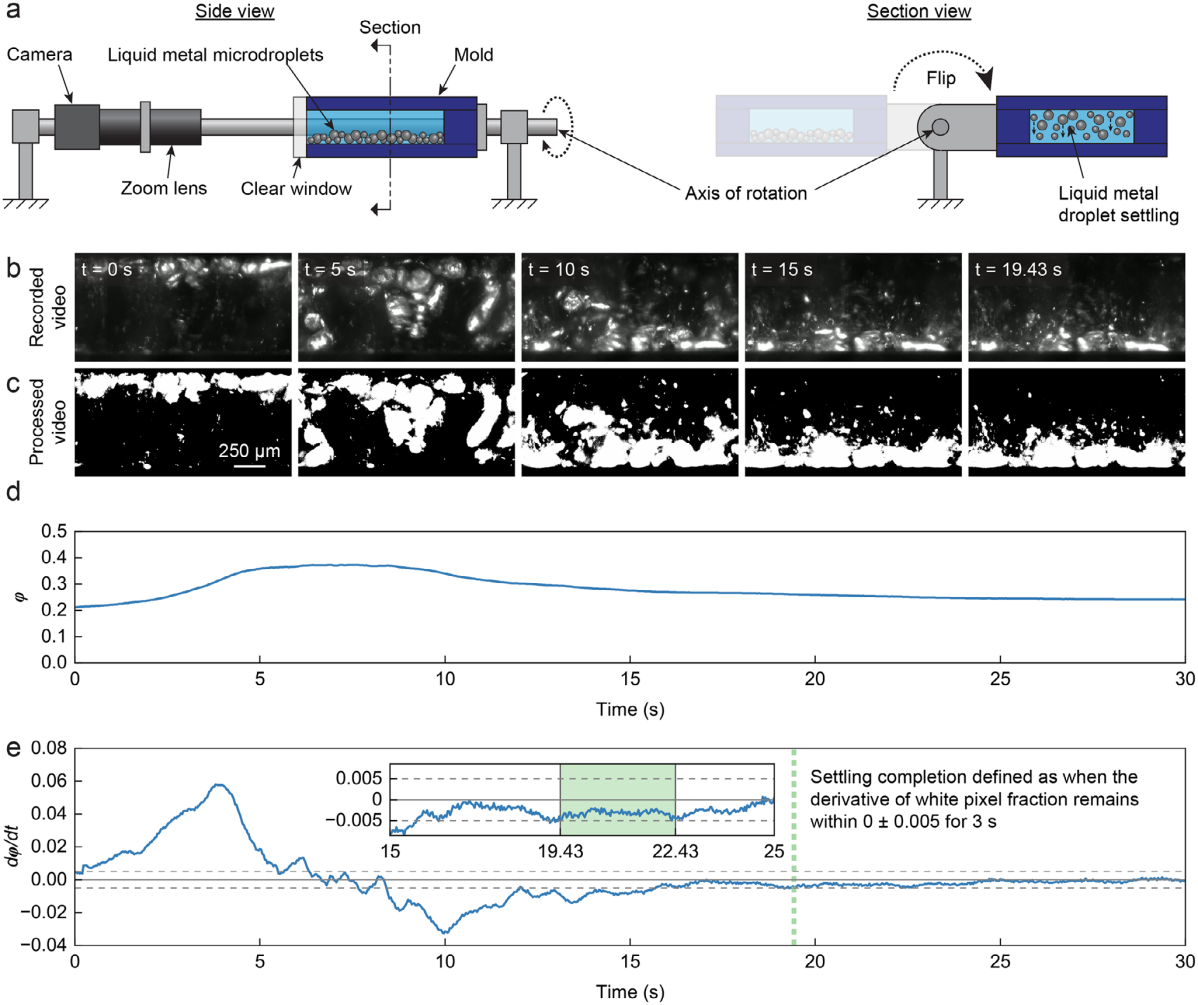
Gravitational settling experimental design. (a) Schematic of the experimental apparatus for video recording of gravitational settling. (b) Sample images from a recorded video of LM gravitational settling. Rightmost image depicts the time at which settling is completed. (c) Binary black/white video conversion of video in (b). (d) Fraction of white pixels (φ) over time as measured using frame‐by‐frame image analysis of (c). (e) Derivative of the fraction of white pixels over time (dφ/dt), showing the cutoff threshold for determination of settling time.

To examine the settling of LM microdroplets in viscous resins, a custom experimental apparatus was constructed to record the settling process. The setup consists of a camera with a high‐magnification lens and a sample mold mounted on a rotating axis, allowing the LM suspension to be inverted during testing (Figure 3a; Figure S3). The mold confines the LM–resin suspension to a uniform film geometry with thicknesses of 320, 760, or 1600 μm (Figure S4). The camera rotates with the sample and continuously records droplet motion during settling (Figure 3b).

The sample mold is inverted once to create the initial bed of settled LM microdroplets, and videos are recorded during a second inversion. Upon inversion, LM microdroplets can be observed to fall from the top surface of the film to the bottom, creating a comparable layer of settled LM microdroplets on the opposite surface (Video S1). This process directly emulates that used in prior works leveraging gravitational settling for LM electronics fabrication [47]. Larger droplets or clumps are frequently observed to settle at greater velocities, with the smallest microdroplets reaching the settled bed last. Further details on sample mold assembly and the inversion process are provided in the Note S1.

To quantitatively characterize the settling behavior, a video‐based image analysis workflow was developed. Each frame was converted to a binary representation that isolates the LM droplets, enabling independent tracking of their motion throughout the film (Figure 3c). The fraction of the image which is primarily LM (i.e., white pixels (φ)) is calculated through image analysis for a region bounded by the top and bottom of the film and the left and right edges of the image frame (Figure 3d). As LM microdroplets transfer from one side of the film to the other, more LM is visible during the settling process than before inversion or after completion. To determine when settling is completed, the derivative over time of the white pixel fraction (dφ/dt) in the video is calculated using MATLAB's diff() function to determine when equilibrium is achieved (Figure 3e). Equilibrium was defined as when the white pixel fraction is within 0±0.005 for a duration of 3 s (Figure 3e inset). These parameters were selected to represent the first point after inversion at which LM microdroplets were motionless and settled to a maximum packing fraction, with the exception of especially small microdroplets that may remain suspended. This provides a single metric of “settling time,” which we use here to compare the effects of various material and process parameters.

### 2.3 Gravitational Settling Analysis

To investigate the impact of material and process parameters on the gravitational settling of LM microdroplets in polymer resins, the input parameters of droplet size, viscosity, LM‐saturated layer thickness, and total film thickness are varied and settling times are measured. In order to employ a framework for understanding the effects of each parameter, we look to Stokes' law [56, 57, 58], which defines the terminal velocity ($u_{T}$) at which a spherical particle can settle in a fluid:

$$u_{T}=\frac{g\;{d_{p}}^{2}\;\left(\rho_{LM}-\rho_{resin}\right)}{18\;\mu }$$

in which g is acceleration due to gravity, $d_{p}$ is the diameter of the spherical inclusion, μ is the viscosity of the suspending fluid, and $\rho_{LM}$ and $\rho_{resin}$ will refer to the densities of inclusion (LM) and the suspending resin, respectively. Stokes' law can also apply to free‐falling droplets in discrete liquid‐liquid suspensions [59], and liquid gallium droplets have been shown to conform to Stokes' law in conditions where near‐sphericity is maintained [60, 61]. The LM‐resin systems investigated here satisfy the low particle Reynolds number (< 0.01) condition for this formula to be useful (see Note S1). For suspensions with many inclusions, Maude and Whitmore [57, 62] provided a modification to account for the hindered settling of packed inclusions:

$$u_{T}=\frac{g\;{d_{p}}^{2}\;\left(\rho_{LM}-\rho_{resin}\right)}{18\;\mu }{\left(1-V_{LM}\right)}^{4.6}$$

in which $V_{LM}$ is the volume fraction of inclusions in the suspension, and the exponent 4.6 is appropriate for low Reynolds numbers. However, while individual, near‐spherical LM microdroplets have been shown to be in alignment with Stokes' law, the settling of highly‐packed LM microdroplets in thin polymer films also includes boundary conditions from the constrained film geometry, dense droplet packing, and non‐uniform size and sphericity of LM droplets, requiring further experimental investigation and comparison.

#### 2.3.1 Settling Time With Varying Microdroplet Size

The settling time ($t_{s}$) for varying LM microdroplet size was measured in a resin mixed to a ratio of 25% by mass high‐viscosity resin and 75% low‐viscosity resin diluent. To align with the diameter term used in Stokes' law, a mean equivalent spherical diameter ($d_{e}$) of LM microdroplets is calculated as:

$$d_{e}={\left(\frac{6\;v_{d}}{\pi }\right)}^{1/3}$$

in which $v_{d}$ is the volume of an LM droplet, assuming a prolate spheroid shape of $v_{d}=4/3\;\pi \;d_{major}\;{\left(d_{minor}\right)}^{2}$ [58]. The mean values of major and minor diameters are used, and equivalent spherical diameters of 180, 148, 107, and 94.6 μm are found for the different mixing parameters, listed from low to high mixing speed and time (Figure S1).

**Figure 4:**
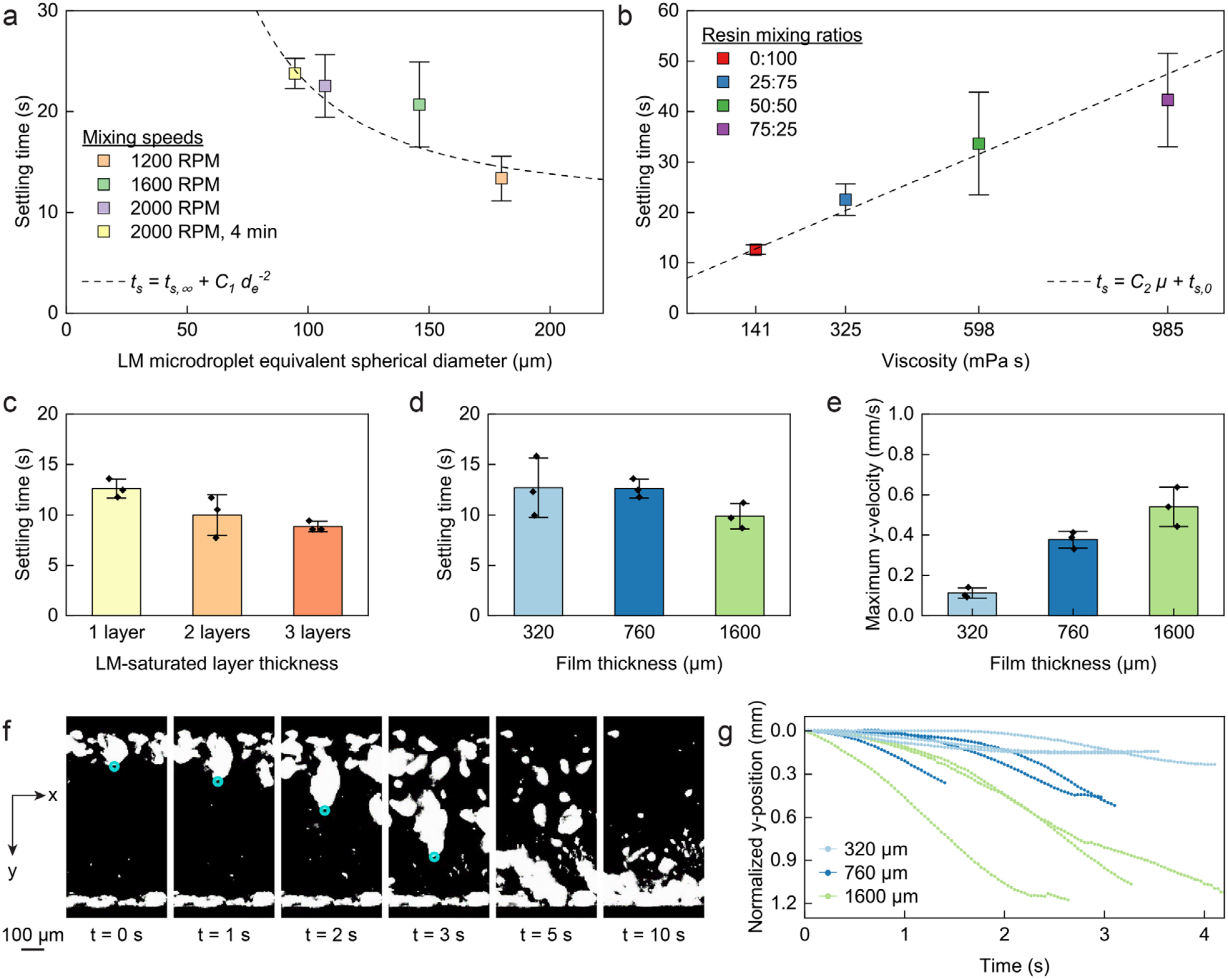
Gravitational settling of LM microdroplets. (a) Settling time for varying sizes of LM microdroplets measured in a 25:75 resin mixing ratio. Coefficients are $t_{s,\infty }=10.9$ s and $C_{1}=119000$ μm ^2^ s, and $R^{2}=0.902$. (b) Settling time for varying viscosities of the suspending resin with while maintaining a constant LM microdroplet size of $d_{e}=107$ μm. Coefficients are $t_{s,0}=6.97$ s and $C_{2}=0.0412$ mPa^−1^, and $R^{2}=0.961$. (c) Settling time varying initial thicknesses of the LM‐saturated layer. (d) Settling time varying the total thicknesses of the film. (e) Maximum velocity in the through‐thickness direction of LM microdroplets for each thickness of film. (f) Sample images of LM microdroplet video motion tracking. Cyan circle represents the tracked point. (g) LM microdroplet position along the through‐thickness direction, normalized to initial position, over time. All data represents mean value ± s.d. (n = 3).

An increase in microdroplet size is shown to cause a decrease in the time required for settling to complete (Figure 4a). This is reflective of the increase in settling velocity of larger particles predicted by Stokes' law as $u_{T}\propto {d_{e}}^{2}$. Therefore, the time required to settle ($t_{s}$) will scale as $t_{s}\propto u_{T}^{-1}\propto {d_{e}}^{-2}$. To fit the experimental data we set $t_{s}=t_{s,\infty }+C_{1}{d_{e}}^{-2}$, where $t_{s,\infty }$ and $C_{1}$ are fitting parameters, which provides a close match to the experimental trend ($R^{2}=0.902$), and captures the $t_{s}\propto {d_{e}}^{-2}$ scaling predicted by the model. Notably, the fitted coefficient $C_{1}$ agrees with the magnitude expected from Stokes' law, which predicts:

$$C_{1}=\frac{18\;\mu \;h}{g\;\left(\rho_{LM}-\rho_{resin}\right)\;{\left(1-V_{LM}\right)}^{4.6}}$$

where h is the total thickness of the film. Using h=760 μm, this yields a calculated value of $C_{1}=86400$ μm ^2^ s if the hindered settling adjustment is forgone, slightly less than the fitted value of 119 000 μm ^2^ s. Assuming a volume fraction of LM microdroplets within the LM‐saturated layer to be that of random loose packed disordered spheres, or $V_{RLP}=55.5$% [63, 64], and having measured the height of the LM‐saturated layer to be $h_{LM}/h=23.1$%, the hindered settling condition predicts a larger value of $C_{1}=162000$ μm ^2^ s for a LM volume fraction of $V_{LM}=V_{RLP}\;h_{LM}/h=12.8$%. This places our experimental fitting parameter between the unhindered and hindered settling model predictions. We attribute this outcome, alongside the necessity of a y‐adjustment term $t_{s,\infty }$ for precise fitting, to confinement and boundary effects within the film impeding droplet motion and limiting attainment of a true terminal velocity following inversion. Despite these confinement‐induced numerical sensitivities, the predicted inverse‐square scaling with droplet diameter remains, providing a quantitative framework to anticipate and design the kinetics of gravity‐directed LM assembly for soft electronics fabrication.

#### 2.3.2 Settling Time With Varying Viscosity

The settling time for varying photoresin viscosity while maintaining a constant LM microdroplet size of $d_{e}=107$ μm was measured. The time required for settling completion is increased at greater viscosity. We observe that settling occurs rapidly in our lowest‐viscosity resin mixture (141 mPa s), finishing in just 12.6 s compared to over 42 s for a higher‐viscosity mixture (985 mPa s) (Figure 4b). Stokes' law shows $u_{T}\propto \mu^{-1}$. Therefore, the time required to settle will scale as $t_{s}\propto u_{T}^{-1}\propto \mu$. To fit the experimental data we set $t_{s}=t_{s,0}+C_{2}\mu$, where $t_{s,0}$ and $C_{2}$ are fitting parameters, which provides a close match to the experimental trend ($R^{2}=0.961$), and captures the $t_{s}\propto \mu$ scaling predicted by the model. $C_{2}$ also aligns to the prediction using Stokes' law hindered settling, as it would be:

$$C_{2}=\frac{18\;h}{g\;{d_{p}}^{2}\;\left(\rho_{LM}-\rho_{resin}\right)\;{\left(1-V_{LM}\right)}^{4.6}}$$

where using $d_{p}=107$ μm and $V_{LM}=12.8$% yields a predicted value of $C_{2}=0.0436$ mPa^−1^, very close to the fitted value of $C_{2}=0.0412$ mPa^−1^.

We note that sufficiently high viscosity has been shown to significantly inhibit the ability of LM to settle in other systems designed for homogeneous dispersions, though it will still occur in media with a viscosity as high as 5000 mPa s [42, 44, 49]. When using only the higher viscosity resin without diluent (μ=1790 mPa s), the time for settling to complete increases to approximately 3 min, taking longer than predicted from the Stokes' law relationship (Figure S5). When evaluating settling in PDMS (μ=5580 mPa s), a widely used matrix material for LM composites, the settling time increases substantially to approximately 1 h. To represent a practical LM composite fabrication workflow, the same mixing and processing conditions were maintained across all resin systems. Under these conditions, the higher viscosity of PDMS produces a finer LM microdroplet dispersion [49], reducing the average droplet diameter to approximately 60 μm. Consequently, the prolonged settling time in PDMS arises from the combined effects of increased matrix viscosity and reduced droplet size, both of which act to decrease settling velocity. Additionally, a greater number of small LM microdroplets remain suspended instead of settling. Therefore, while the linear increase in setting time as a function of viscosity is captured within the primary range of viscocities examined, extreme values of viscosity are expected to deviate from this prediction as droplets become homogeneously suspended.

#### 2.3.3 Settling Time With Varying Film Parameters

Variations in both total film thickness and LM‐saturated layer thickness produce comparatively smaller changes in settling time than droplet size or viscosity. When the thickness of the LM‐saturated layer, and thus also the overall volume fraction of LM, was increased by using one, two, or three layers of adhesive mask during drawdown, settling time decreased modestly from 12.6 s for a single layer to 8.86 s for three layers (Figure 4c). As the overall film thickness was held constant, this reduction is most likely attributable to the shorter travel distance required for each microdroplet. Notably, even in the thickest three‐layer configuration, droplets settle into a similarly uniform bed as observed in the single‐layer samples (Figure S2d,e).

Varying the total film thickness also resulted in only modest changes in settling time (Figure 4d), with thicker films exhibiting a slight decrease that remains largely within experimental error. However, video motion tracking reveals a notable increase in maximum droplet velocity with increasing film thickness (Figure 4e), reaching 0.54 mm/s in 1600 μm films – approximately five times greater than in 320 μm films. With microdroplet size and viscosity remaining the same, this suggests that terminal velocity may not be reached in the shorter settling distance of thinner films. Individual droplet trajectories obtained through video motion tracking show that settling in thicker films initiates and progresses more rapidly (Figure 4f), such that droplets in all films complete settling over approximately the same duration regardless of total thickness (Figure 4g).

These observations indicate that confinement and wall effects imposed by the film geometry strongly influence the settling dynamics. The increase in peak microdroplet velocity with greater film thickness is consistent with classical hydrodynamic confinement effects, where droplets in thinner films remain in close proximity to confining surfaces throughout much of the settling process, increasing viscous resistance relative to unbounded Stokes settling through wall‐mediated hydrodynamic drag [65, 66, 67]. As film thickness increases, droplets experience a larger central region with reduced wall influence, enabling higher peak settling velocities. Despite this increase in velocity, the overall settling time changes only modestly because the greater travel distance in thicker films partially offsets the enhanced droplet mobility. Together, these observations suggest a transition from strongly confined toward more bulk‐like settling behavior as the confinement ratio, ($d_{e}/h$), decreases, where ($d_{e}$) is the droplet diameter and (h) is the film thickness. As a result, total settling time is only weakly dependent on film thickness, affording flexibility in film design while leveraging gravitational settling for LM circuit fabrication.

### 2.4 Settling in Soft, LM Interlayer Vias

**Figure 5:**
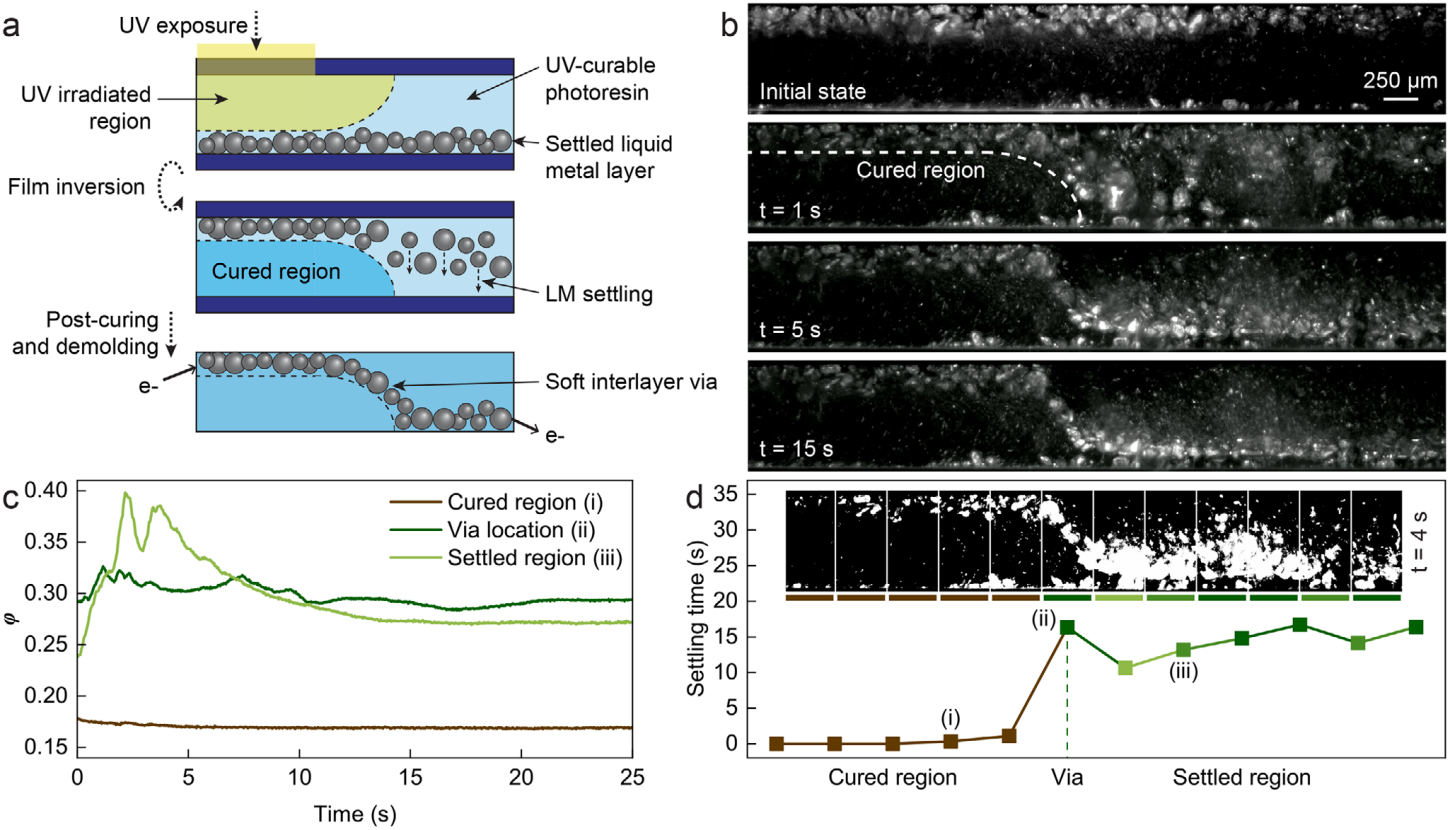
LM settling for soft interlayer vias. (a) Schematic of the LM‐STAIR fabrication process. (b) Settling of LM with a region of cured resin. (c) Fraction of white pixels (φ) over time for various sections across the film including the via, the region in which curing occurs, and the region in which settling occurs. (d) Settling time for discrete sections of the film. Inset shows image sections corresponding to each data point, and (i), (ii) and (iii) indicate data corresponding to plots in (c).

One application of gravitational settling of LM in polymer resin films is the fabrication of interlayer vertical interconnect accesses (vias) within multilayer LM circuits. The “liquid metal stratification for three‐dimensional (3D) assembly of electrical interconnects (LM‐STAIR)” fabrication process uses LM microdroplet setting alongside selective curing of a photo‐curable polymer matrix to create soft and stretchable vias [47]. UV light is patterned through a shadow mask to cure designated regions (Figure 5a), and the use of a non‐collimated light source produces a graded cure profile with curved interfaces that define via formation sites. Upon inversion, LM microdroplets in the uncured regions settle toward the opposite surface, accumulating along the edges of cured domains to form conductive vertical interconnects (Figure 5b). This creates an electrical circuit with vias that can reliably conduct electricity between multiple layers, enabling more complex and advanced circuit designs. These LM interconnects and vias have been shown to possess robust electrical stability under many conditions, including while under strain, for varying film thicknesses, and in a variety of photoresin compositions with varying viscosity [47]. As settling and curing occur simultaneously across the entire film, all vias are assembled in parallel, offering a scalable alternative to sequential via fabrication.

Using the experimental platform and video‐processing framework developed here, the movement of LM microdroplets in the cured region, via, and settled region are selectively analyzed. In the cured region, the fraction of white pixels (φ) undergoes negligible change, indicating the LM microdroplets have been fixed in position by the selective UV curing process (Figure 5c). At the via interface, the magnitude of change in φ is reduced relative to fully uncured regions, yet the characteristic settling duration remains comparable, indicating that droplet transport kinetics are largely preserved even near partially cured boundaries. Total settling times in the uncured region are relatively consistent regardless of the distance from the via, indicating that the generalizations drawn for the settling of bulk films are transferrable directly to patterned, fabrication‐relevant geometries. (Figure 5d). Furthermore, we measure traces of this material system to have a conductivity of $\sigma =1.3\times 10^{5}$ S m^−1^, based on the measured resistance R=2.1±0.25Ω of a trace with length l=50 mm, width w=1 mm, thickness t=176 μm, and $\sigma =\frac{1}{R}\frac{l}{wt}$ (Figure S6). These results establish that gravitational settling can be quantitatively predicted and spatially confined within structured curing fields, providing a mechanistic basis for process optimization and scalable manufacturing of multilayer soft electronic architectures.

## 3 Conclusion

Gravitational settling of LM microdroplets provides a scalable pathway for fabricating soft and stretchable circuits, with settling kinetics governed primarily by droplet size and, most strongly, by resin viscosity. Increasing droplet diameter and decreasing matrix viscosity accelerate assembly, while variations in film thickness or saturated layer thickness show only minor influence on total settling time. This weak dependence on through‐thickness dimensions enables design flexibility for multilayer architectures without compromising fabrication speed.

By establishing quantitative relationships between material properties and settling dynamics, this work provides a predictive framework for process optimization in gravity‐directed LM assembly. These trends are further captured in structured films, showing applicability to a wide range of circuit architectures. The analysis methodology introduced here is readily extensible to emerging challenges, including multiphase inclusions, nanoscale LM droplets, graded architectures, and multistep additive manufacturing processes. Beyond soft electronics, these insights inform broader classes of gravity‐driven structuring phenomena in composite materials, thin films, and reactive resins, where confinement, interfacial interactions, and curing kinetics interact with particle transport. Together, this understanding advances the scalable manufacturing of conformal electronics while contributing to the fundamental science of confined multiphase flow and directed assembly in soft materials systems.

## 4 Methods

### 4.1 Material Preparation

Eutectic gallium–indium (EGaIn) liquid metal was made by combining a 3:1 by mass ratio of gallium and indium on a hot plate at 200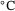 overnight. Resins were prepared by hand‐mixing a low‐viscosity diluent resin (Ebecryl 113) and a high‐viscosity resin (Resione F39T), and PDMS (Sylgard 184) was used for highest viscosity resin. Black dye (Alumilite Black Opaque Resin Dye) was added to approximately 0.02% by mass to aid in contrast. EGaIn LM was dispersed in resin (40% LM by volume) using a dual asymmetric centrifugal mixer (Flacktek DAC 1200–500 VAC SpeedMixer) at either 1200, 1600, or 2000 RPM for 2 min, or at 2000 RPM for 4 min.

### 4.2 Optical Microscopy

Top down optical microscope images were obtained using a Zeiss Axio Zoom v16 stereo microscope with sample images taken in three different regions for each specimen.

### 4.3 LM Microdroplet Analysis

The Python OpenCV library was used to detect droplet outlines in microscope images. Contours in the image were detected and drawn onto the microscope image, then manually edited to reduce noise and overlapping borders. Using Fiji software, microscope images were binarized along the contours, and ellipses were fit onto each droplet. The major and minor diameters were calculated for each ellipse.

### 4.4 Rheological Analysis

Apparent viscosity of polymer resin mixtures was measured using a rheometer (TA Instruments Discover HR‐30) with a 40 mm parallel‐plate fixture. Mixing ratios of 0:100, 25:75, 50:50, 75:25, and 100:0 by mass of low‐viscosity diluent resin to high‐viscosity resin were measured, as well as PDMS without crosslinking agent. A steady flow sweep test was run with shear rates from $10^{-1}$ to $10^{3}$ s^−1^. Data points with a torque measurement less than 3 μN m were omitted. Measurements were conducted at room temperature.

### 4.5 Gravitational Settling Characterization

Dispersions of EGaIn LM in resin were cast onto a substrate with an adhesive mask, and the suspension was drawn down to the thickness of the mask with a plastic razor. The remaining pieces of the film geometry mold were assembled and filled with resin, and the assembly is mounted to the custom experimental apparatus. The experimental apparatus includes a USB camera (FLIR Blackfly S) and a high‐zoom lens (Edmunds Optics VZM 450i) mounded to a purpose‐built frame (Figure S3). The assembly is rotated 180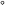 and videos are recorded using the FLIR Spinview software. Total settling time is determined through the video analysis framework described. For samples with long settling durations, settling time was determined through visual inspection. Additional details on sample preparation are provided in the Sample Assembly section of Note S1 and Figure S4, and additional details on the experimental process are provided in the Film Inversion Procedure section of Note S1.

### 4.6 Video Motion Tracking of Individual LM Microdroplets

The binary format black or white videos used in settling analysis were loaded into a video motion tracking software (Physlets Tracker). Tracking points were assigned at the initial frame, and an automatic tracking function was employed to track motion without using any manual location selection. Tracking points were abandoned and a new initial location was selected if the automatic tracking function failed.

### 4.7 Selective UV Curing

Experiments with UV curing alongside settling of LM microdroplets were based on the LM‐STAIR process [47]. One side of the mold used in settling experiments was replaced with clear acrylic, and an opaque film was cut to shape and used as a shadow mask. A UV light panel was mounted above the sample in the experimental apparatus and turned on for 20 s. The film was then inverted in the experimental apparatus as previously described.

## Author Contributions

B.T.W. and M.D.B. conceived the idea; B.T.W. designed experiments with input from R.C.R. and M.D.B.; B.T.W. and R.C.R. performed experiments; B.T.W. and R.C.R. analyzed data; B.T.W. and M.D.B. wrote the paper with input from R.C.R.; and M.D.B. supervised the study.

## Conflicts of Interest

The authors declare no conflicts of interests.

## Supporting information


**Supporting File 1**: smll74498‐sup‐0001‐SuppMat.pdf.

**Supporting File 2**: smll74498‐sup‐0002‐VideoS1.mp4.

**Supporting File 3**: smll74498‐sup‐0003‐VideoS2.mp4.

## Data Availability

Data and computer code available on request from the authors.
