# Supplementary material for: Gravity‐Driven Assembly Dynamics of Liquid Metal Microdroplets for Functional Composite Films
Source: Small. 2026 Jul 13;22(49):e74498. doi: 10.1002/smll.74498 (PMC13533223; doi:10.1002/smll.74498)
Supplement: Supplementary file 1 [file SMLL-22-e74498-s001.pdf]

Supplementary Information

Gravity-driven assembly dynamics of liquid metal microdroplets for functional composite films

Brittan T. Wilcox[1], Ryan C. Rothermel[1], and Michael D. Bartlett[1,2*]

[1]Mechanical Engineering, Soft Materials and Structures Lab, Virginia Tech, Blacksburg, VA 24061, USA.

[2]Macromolecules Innovation Institute, Virginia Tech, Blacksburg, VA 24061, USA.

[*]Corresponding author email: mbartlett@vt.edu

Supplementary Notes

Sample Assembly

13 g of LM and 3 g of resin (40% LM by volume) were combined in a 10 mL mixing cup and mixed in a dual asymmetric centrifugal mixer (Flacktek DAC 1200–500 VAC SpeedMixer) at 1200, 1600, or 2000 RPM for 2 minutes or at 2000 RPM for 4 minutes.

Assembly of the sample mold is shown in Figure S4. An adhesive mask (Blazer Orange Laser Mask, Johnson Plastics Plus) was applied to the surface of a 1/8" thick cast acrylic sheet (McMaster #8505K742) using a roller. Additional layers of adhesive mask were added for experiments varying the thickness of the LM layer. This was laser cut (Universal Laser Systems VLS4.75) using two different parameters to selectively cut either adhesive mask only or both mask and acrylic sheet simultaneously. A 10 mm x 10 mm cut square of adhesive mask was removed and the LM-resin solution was cast liberally onto the surface.After 30 s, excess solution was scraped away with a plastic razor, with the remaining material being solely a settled and highly packed solution of LM microdroplets in resin. The remaining adhesive mask was removed.

Polydimethylsiloxane (DOW Sylgard 184) was combined at a 20:1 weight ratio of base to curing agent, fully mixed in a dual asymmetric centrifugal mixer, cast to a thickness of 500 µm on a clear 1/8" thick cast acrylic sheet (McMaster #8560K257) using a film applicator, fully cured, then laser cut to shape. Additional mold pieces were laser cut from 1/8" cast acrylic sheet (McMaster #8505K742), 1/16" (1600 µm) acrylonitrile butadiene styrene sheet (McMaster #8586K151), 0.030" (760 µm) plastic shim stock (McMaster #9513K25), or 0.0125" (320 µm) plastic shim stock (McMaster #9513K21).

Sample containers were assembled and clamped together with binder clips. Unfilled resin was added on top of the cast LM-resin solution to fill the film mold of the sample container. The assembly is sealed, then affixed to the filming fixture with a bolt.

Film Inversion Procedure

After the sample container is affixed to the gravitational settling experimental apparatus, an initial flip is performed. All flips are performed by hand using a smooth, controlled motion, and an approximately 2 mm thick sheet of neoprene foam installed on the resting plate for the fixture prevents harsh impacts upon placement in the final inverted position. Due to LM microdroplets being caught at the edge of the mold used in casting and a groove resulting from the laser cutting process, some LM microdroplets in view of the camera remain adhered to the initial surface and either do not settle, or take an extended period to release from the surface before settling. To accurately characterize the settling behavior of the bulk of the LM microdroplets, a second flip (retuning to the initial position) is performed. All gravitational settling data represented in this work is taken on this second flip.

Particle Reynolds Number

The applicability of Stokes' law requires, among other conditions, a particle Reynolds number less than 0.01.[58] Particle Reynolds number is calculated through the equation

$$Re_p = \frac{d_p \, u \, \rho_{resin}}{\mu}$$

in which d_p is the particle diameter, u is the velocity, ρ_{resin} is the density of the resin, and μ is the resin viscosity. Assuming the values which would allow the greatest particle Reynolds number, we will establish that the upper bound of possible particle Reynolds numbers is less than the limiting condition of 0.01. For droplet diameter, no major nor minor diameters of droplets were measured in excess of 1 mm, with very few exceeding 500 µm (see Fig. 2c and S1). The greatest settling velocity measured using motion tracking analysis was 0.638 mm/s (See Fig. 4e). Of the two polymer resins used, the greater density is 1079 kg/m^3. The lowest viscosity measured was 0.141 Pa s (See Fig. 2d). With these, the highest possible particle Reynolds number would be

$$Re_p = \frac{(0.001\ \text{m})(6.38 \times 10^{-4}\,\text{m/s})(1079\ \text{kg/m}^3)}{(0.141\ \text{Pa s})} = 0.00488$$

and it is thus reasonable to assume that the particle Reynolds number for any experimental condition in this work is less than 0.01.

Supplementary Figures

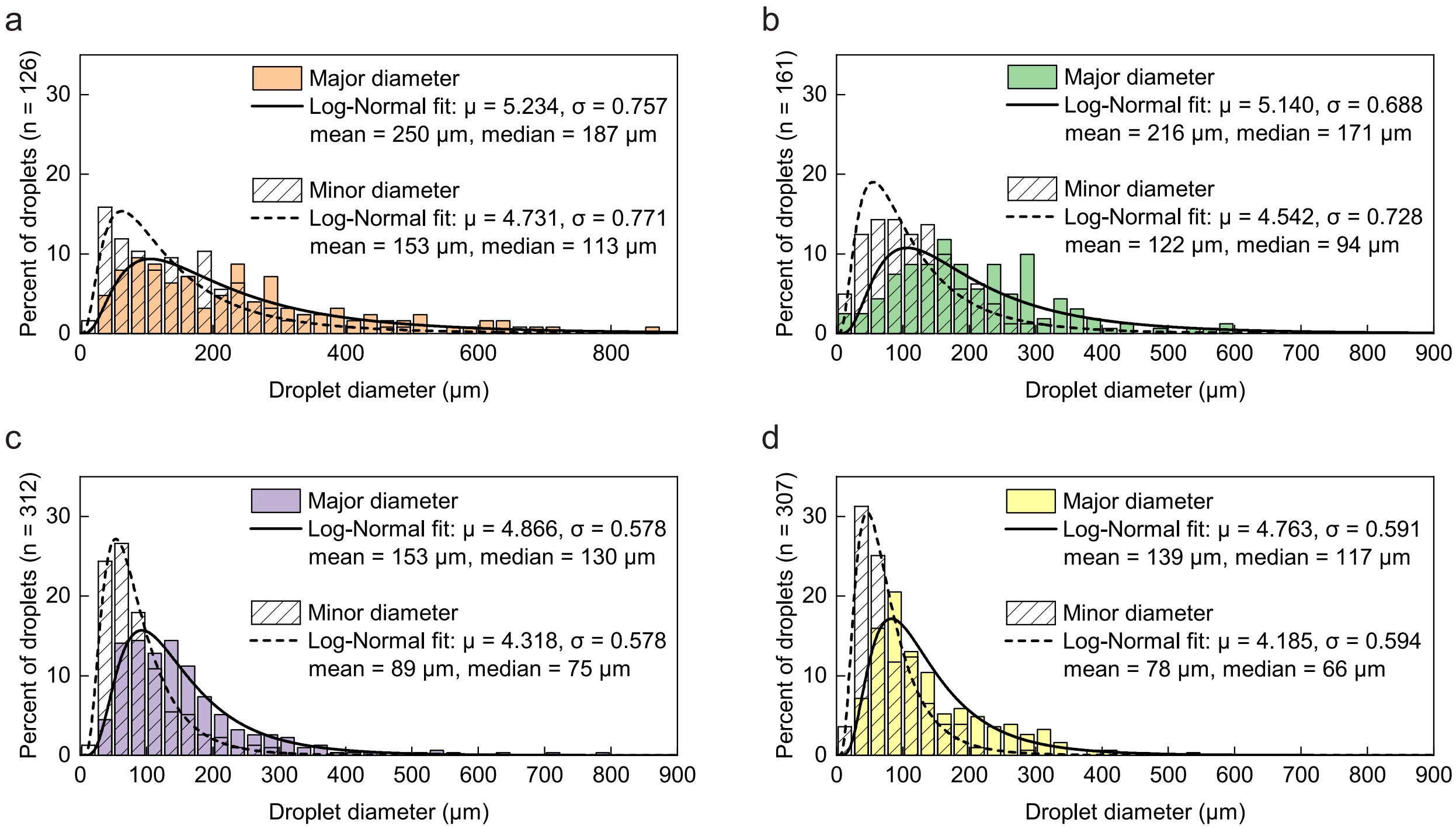

Fig. S1 Droplet analysis. Histograms of droplet major and minor diameters for mixing speeds of **a)** 1200 RPM for 2 minutes, **b)** 1600 RPM for 2 minutes, **c)** 2000 RPM for 2 minutes, and **d)** 2000 RPM for 4 minutes. Mean and median values were calculated for each dimension by the formulae $e^{\mu+\frac{\sigma^2}{2}}$ and e^{μ}, respectively.

Mixing speed	Mixing time	Dimension	Log-Normal fit		Statistical parameters		Eq. spherical diameter	d_{10}	d_{50}	d_{90}
			μ	σ	mean	median				
RPM	min				µm	µm	µm	µm	µm	µm
1200	2	Major	5.234	0.757	250	187	180	65.8	217	490
		Minor	4.731	0.771	153	113		35.4	134	273
1600	2	Major	5.140	0.688	216	171	148	76.3	186	349
		Minor	4.542	0.728	122	94		38.9	107	210
2000	2	Major	4.866	0.578	153	130	107	61.8	131	276
		Minor	4.318	0.578	89	75		36.1	72.1	169
2000	4	Major	4.763	0.591	139	117	94.6	56.5	113	272
		Minor	4.185	0.594	78	66		30.0	64.3	147

Table S1: Statistical and fitting parameters for LM droplet diameters from each mixing condition.

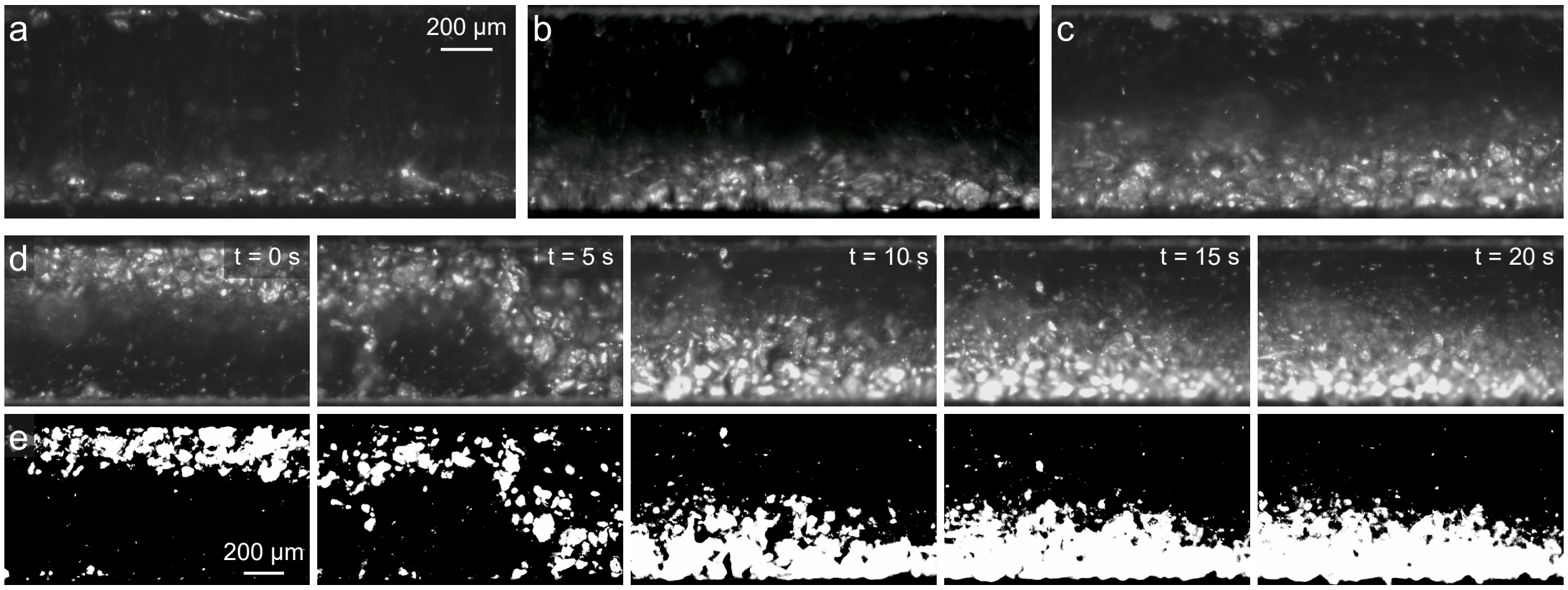

Fig. S2 Settling of thicker LM-saturated layers. Horizontal section micrographs of settled LM-saturated layers cast over **a)** 1 layer of adhesive mask, **b)** 2 layers of adhesive mask, and **c)** 3 layers of adhesive mask. **d)** Settling of a 3-layer sample and **e)** corresponding binary format black and white video.

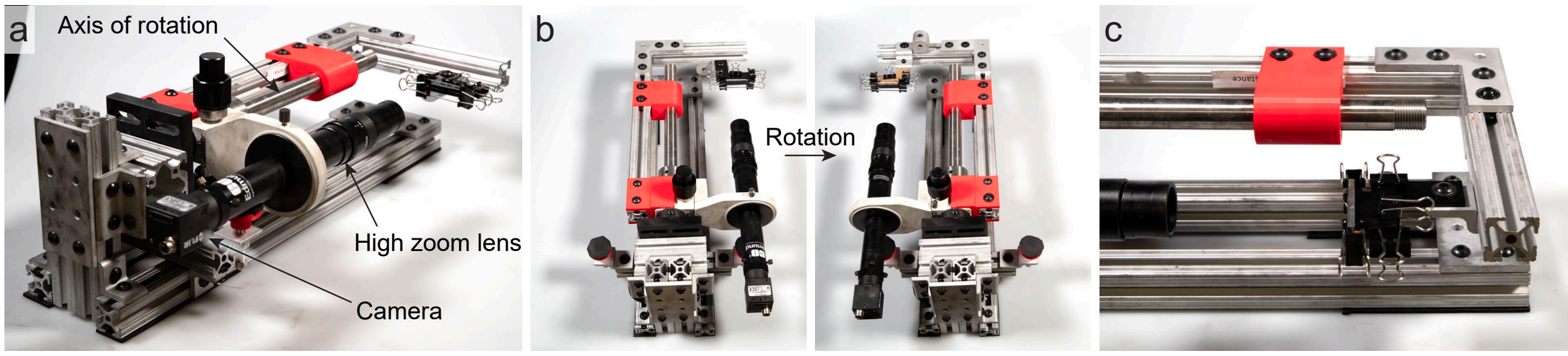

Fig. S3 Gravitational settling experimental apparatus. **a)** Isometric view of experimental apparatus with labels. **b)** Overhead view of apparatus in both an initial and flipped state. **c)** Sample mold assembly mounted to experimental apparatus.

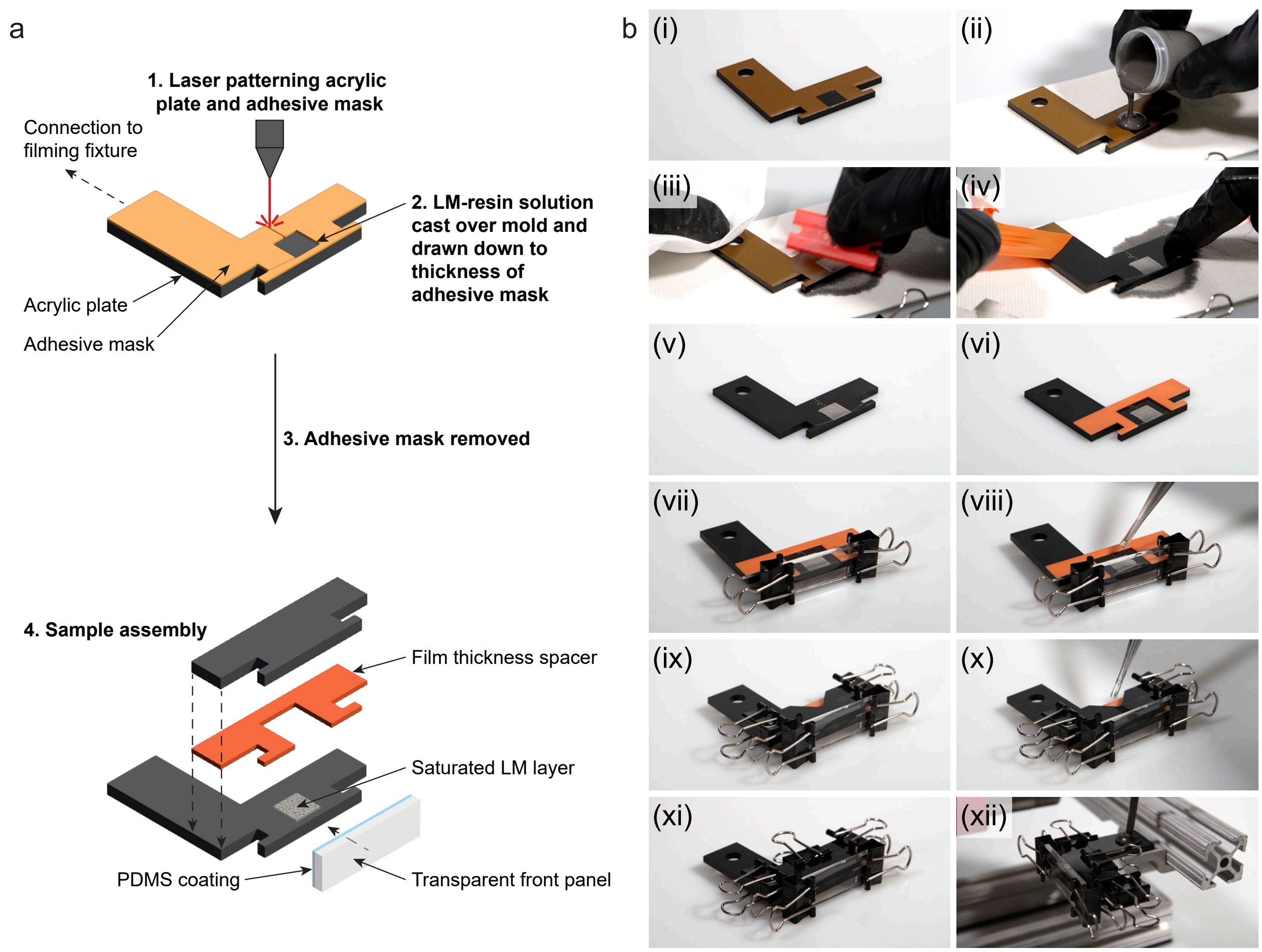

Fig. S4 Design of sample mold. a) Schematic of sample mold assembly with process steps. **b)** Images of sample mold assembly process, including (i) laser-patterned plate and adhesive mask, (ii) casting LM-resin solution, (iii) drawdown of LM-resin solution, (iv) removal of adhesive mask, (v) remaining LM-saturated layer, (vi) placement of spacer to set film thickness, (vii) attachment of transparent front panel with clips, (viii) addition of unfilled polymer resin, (ix) attachment of top plate with clips, (x) final filling of resin, (xi) final sealing of mold, and (xii) attachment of sample mold to experimental apparatus.

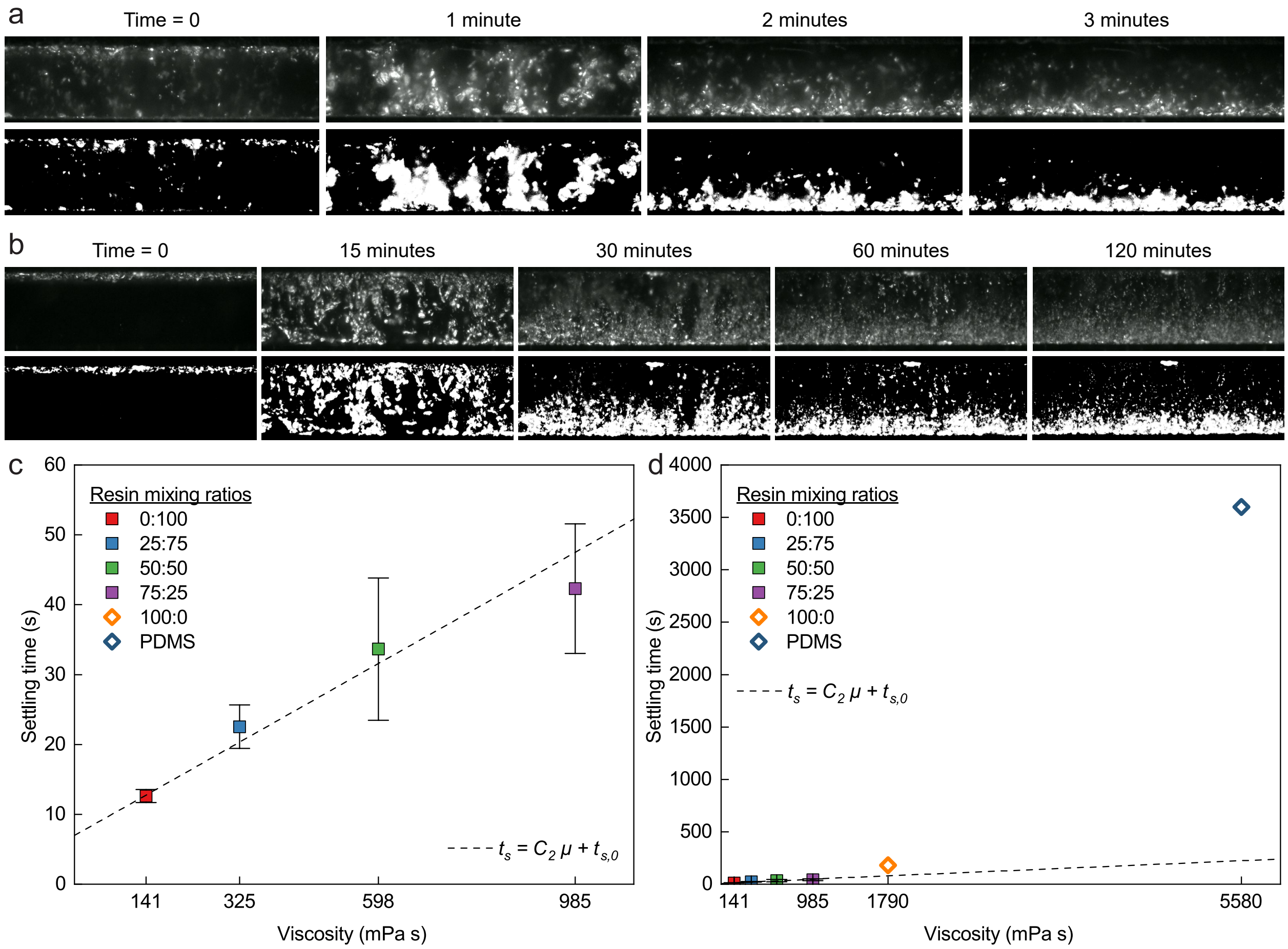

Fig. S5 Settling with higher viscosity. **a)** Images of settling using only the higher viscosity resin without diluent (μ = 1790 mPa s). **b)** Images of settling using uncured PDMS (μ = 5580 mPa s). **c)** Settling time for varying viscosities over the primary range of interest of the suspending resin with while maintaining a constant LM microdroplet size of d_e = 107 µm. Coefficients are $t_{s,0}$ = 6.97 s and C_2 = 0.0412 mPa^{-1}, and R^2 = 0.961. **d)** Approximate settling time for varying viscosities over an extended range with the same fitting coefficients of $t_{s,0}$ = 6.97 s and C_2 = 0.0412 mPa^{-1}. For the PDMS (μ = 5580 mPa s), the higher viscosity of PDMS produces a finer LM microdroplet dispersion, reducing the average droplet diameter to approximately 60 µm. Consequently, the prolonged settling time in PDMS arises from the combined effects of increased matrix viscosity and reduced droplet size, both of which act to decrease settling velocity.

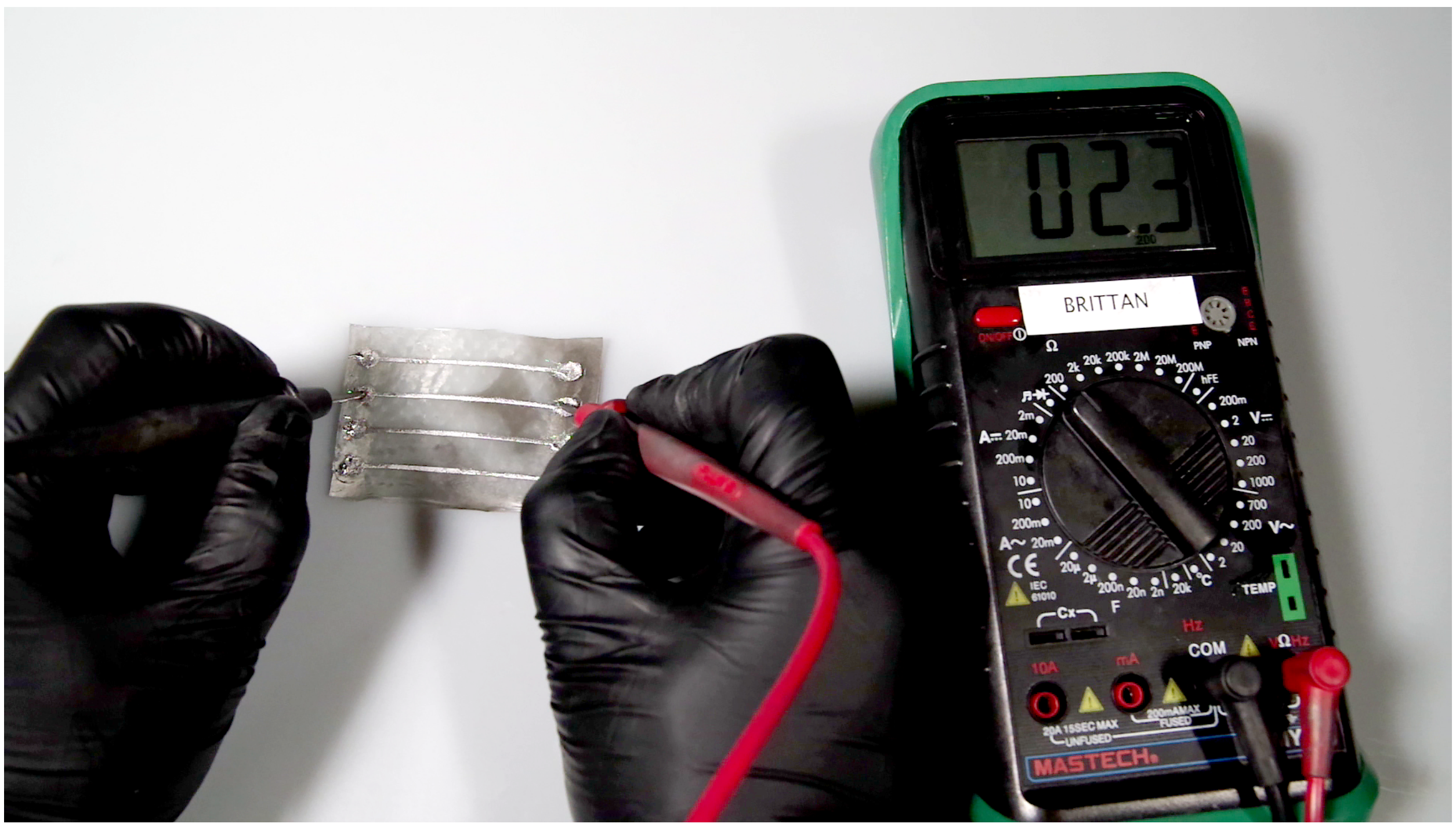

Fig. S6 LM composite trace conductivity.
